# Neutralizing Antibodies to Human Cytomegalovirus Recombinant Proteins Reduce Infection in an Ex Vivo Model of Developing Human Placentas

**DOI:** 10.3390/vaccines10071074

**Published:** 2022-07-04

**Authors:** Takako Tabata, Matthew Petitt, Julia Li, Xiaoyuan Chi, Wei Chen, Irina Yurgelonis, Sabine Wellnitz, Simon Bredl, Tiago Vicente, Xinzhen Yang, Philip R. Dormitzer, Lenore Pereira

**Affiliations:** 1 Cell and Tissue Biology, School of Dentistry, University of California, San Francisco, CA 94143, USA; takakotabata906@gmail.com (T.T.); petitt@mac.com (M.P.); 2 Vaccine Research and Development, Pfizer, Inc., New York, NY 10965, USA; julia.li@pfizer.com (J.L.); schi01@yahoo.com (X.C.); wei.chen12@pfizer.com (W.C.); irina.yurgelonis@pfizer.com (I.Y.); sabine.wellnitz@pfizer.com (S.W.); xinzhen.yang.6561@gmail.com (X.Y.); bvirol@gmail.com (P.R.D.); 3 RedVax Inc., a Wholly Owned Subsidiary of Pfizer, Inc., 8001 Zurich, Switzerland; simon.bredl@gmail.com (S.B.); tvicente@gmail.com (T.V.)

**Keywords:** cytomegalovirus, vaccine, congenital infection, placenta

## Abstract

Human cytomegalovirus (HCMV) is the leading viral cause of congenital disease and permanent birth defects worldwide. Although the development of an effective vaccine is a public health priority, no vaccines are approved. Among the major antigenic targets are glycoproteins in the virion envelope, including gB, which facilitates cellular entry, and the pentameric complex (gH/gL/pUL128-131), required for the infection of specialized cell types. In this study, sera from rabbits immunized with the recombinant pentameric complex were tested for their ability to neutralize infection of epithelial cells, fibroblasts, and primary placental cell types. Sera from rhesus macaques immunized with recombinant gB or gB plus pentameric complex were tested for HCMV neutralizing activity on both cultured cells and cell column cytotrophoblasts in first-trimester chorionic villus explants. Sera from rabbits immunized with the pentameric complex potently blocked infection by pathogenic viral strains in amniotic epithelial cells and cytotrophoblasts but were less effective in fibroblasts and trophoblast progenitor cells. Sera from rhesus macaques immunized with the pentameric complex and gB more strongly reduced infection in fibroblasts, epithelial cells, and chorionic villus explants than sera from immunization with gB alone. These results suggest that the pentameric complex and gB together elicit antibodies that could have potential as prophylactic vaccine antigens.

## 1. Introduction

Human cytomegalovirus (HCMV) is the most common viral infection transmitted during pregnancy and is a cause of severe disease outcomes in newborns [1,2,3]. Permanent birth defects are most frequent following primary maternal infection in the first trimester of gestation. These infections can lead to intrauterine growth restriction (IUGR) and fetal impairment from viral replication in the placenta and developing organs or hypoxia associated with placental pathology and brain damage [1,2,4,5,6,7,8,9]. Protection against maternal-fetal transmission depends on developing neutralizing antibodies to the viral glycoproteins and on cell-mediated immunity. Delayed T-cell responses, reduced antibodies to key viral proteins, and low antibody avidity after primary infection are associated with an increased risk of transmission [1,10,11,12,13,14].

In cases of congenital infection and transmission, viral inclusions in placentas are found in trophoblast progenitor cells (TBPCs), cytotrophoblasts (CTBs), villus stromal fibroblasts, and epithelial cells of amniotic membranes at delivery [9,15,16,17]. In early gestation, CTB cell columns that anchor the placenta to the uterine wall are the main targets of infection, suggesting a conduit for virus dissemination [18,19,20,21,22,23]. Infection of these cells could also undermine placental development and lead to pathology [8,9,15,16]. Viral proteins required for attachment and entry vary depending on the target cell. Entry into fibroblasts and epithelial and endothelial cells is blocked by neutralizing antibodies of different specificities [24,25,26,27]. While infection of fibroblasts depends on the envelope glycoprotein gB and complexes gM/gN and gH/gL, infection of epithelial and endothelial cells requires the proteins pUL128, pUL130, and pUL131A, which form a pentameric complex with gH/gL [24,28]. A third complex, composed of gH/gL/gO, facilitates entry into fibroblasts [29]. The pentameric complex is a target of potent neutralizing antibodies in HCMV-seropositive individuals [27,30,31,32,33].

Studies with intravenous hyperimmune globulin (HIG) have suggested the importance of HCMV-specific antibodies in preventing congenital disease. HIG administered to women with primary infection in the first or early second trimester significantly reduced virus transmission [34,35,36,37,38] and placental pathology [8], although better-controlled studies are needed. Assays of amniotic fluid following HIG administration showed reduced HCMV DNA copy numbers and infectivity, in accordance with a lower viral load in the fetal compartment [39]. The depletion of pentameric complex-specific antibodies in HIG significantly reduces the neutralizing capacity, suggesting they play a key role in protection [40,41].

Studies using human monoclonal antibodies (mAbs) demonstrated the capacity of gB- and pentamer-specific antibodies to neutralize HCMV infection of various placental cells. Infection of TBPCs from chorionic membranes and stromal fibroblasts from the villus core can be effectively blocked by mAbs to gB, but not mAbs to pUL128-131 of the pentamer complex [42,43]. In addition, gB-specific mAbs reduce infection of CTBs in anchoring villus explants from early gestation placentas [44]. Nonetheless, pUL128-131-specific mAbs efficiently block infection of primary amniotic epithelial cells (AmEpCs) from amniotic membranes [15,43] and potently reduce infection and spread in CTB cell columns of villus explants [43]. The predominant activities of mAbs generated from B-cells of immunized animals and seroimmune individuals are directed to the pentamer complex and neutralize infection in epithelial and endothelial cells, macrophages, and amniotic epithelial cells [31,43,44,45,46,47,48,49]. The pentamer complex elicits high-titer epithelial and endothelial cell-specific neutralizing antibody responses that are less effective against fibroblast entry, consistent with the neutralizing antibody response induced during natural infection [9,27,30,31,32,33].

The development of effective vaccines can be advanced by a detailed understanding of the capacity of neutralizing antibodies to HCMV proteins to limit maternal-fetal virus transmission during early gestation. Here, we used primary placental cells and an ex vivo model of developing human placentas to determine whether antisera from rabbits and rhesus macaques immunized with HCMV pentamer and gB reduce infection and virus spread. These results help define which vaccine components may be most effective at preventing transplacental transmission during pregnancy.

## 2. Materials and Methods

### 2.1. Antibody Production in Rabbits and Rhesus Macaque

Synthetic recombinant HCMV proteins were obtained from two sources. One set of proteins—the pentameric complex proteins gH, gL, pUL128, pUL130, and pUL131a and the envelope glycoprotein gB—was obtained from Redbiotec (Schlieren, Switzerland; referred to as RBT pentamer and RBT gB). These proteins were expressed using a baculovirus expression vector (BEV) in VE2 insect cells (Oxford Expression Technologies, Oxford, UK) and purified via a C-terminal 8xHis tag. To express the five pentameric complex proteins using one BEV, the five individual genes (based on the Towne strain sequences) were synthesized with codon optimization for insect cell expression without modification, except the gH gene, which was truncated to be expressed as the soluble ectodomain only with 8xHis tag added at the C-terminus for the synthesis of gB. The Towne sequence was codon-optimized for insect cell expression with the gB transmembrane domain removed and a His tag added at the C-terminus. VE2 cells were infected with the corresponding baculovirus (preferably with an MOI > 5 [VPC > 10]) and tagged HCMV proteins were purified from conditioned media using Ni-NTA Sepharose FF columns (GE Healthcare), washed, and eluted with Tris-buffered saline (TBS), pH 7.8, containing increasing concentrations of imidazole (25 mM Tris, 150 mM NaCl, 3 mM KCl, 0–500 mM imidazole). Fractions containing the target products were pooled and diluted in TBS, pH 7.4, to a final imidazole concentration of 75 mM. The resulting solutions were subjected to anion exchange chromatography using a Sartobind Q SingleSep column (Sartorius, Göttingen, Germany), working in negative mode, to remove impurities. The target proteins in the flow-through were further purified on a Chelating Sepharose FF resin column dialyzed in TBS, pH 7.4, to remove imidazole, concentrated by ultrafiltration, and stored at −80 °C. Purified recombinant gB was purchased from Sino Biologicals (Wayne, PA; referred to as Sino gB); its sequence was also based on the Towne strain with the transmembrane domain removed and an 8xHis tag added at the C-terminus. Sino gB also contained an engineered furin cleavage site, TTQT, replacing the sequence RTKR in the original Towne gB amino acid sequence, similar to the gB used in the clinical studies by Sanofi [50], and was produced in HEK293 cells.

### 2.2. Immunization

Rabbits were immunized at Pfizer (Pearl River, NY, USA). Five female New Zealand White rabbits weighing approximately 2.5 kg were immunized with 100 µg of the RBT pentamer and 50 µg QS-21 (Pfizer, Groton, CT, USA) per dose by IM injection at weeks 0, 4, and 8. Sera collected from week 0 (pre-immune, seronegative for HCMV) and weeks 4, 6, 8, and 9 were used for the study. The immunization of male rhesus macaques approximately five years of age was carried out at the New Iberia Research Center (Lafayette, LA, USA). These monkeys were seropositive for rhesus CMV (RhCMV) at baseline. Five animals were immunized with 20 µg of HCMV Sino gB, and five animals were immunized with 20 µg of RBT gB and 100 µg of RBT pentamer, both with 50 µg of QS-21 adjuvant per dose, by IM injection at weeks 0, 2, and 4. Preimmune sera and sera collected at week 6 were used for the study.

### 2.3. Cells and Human Placentas

Studies on HCMV infection of human placentas were approved by the Institutional Review Board of the University of California, San Francisco. Placentas were obtained from uncomplicated deliveries at UCSF Mission Bay Hospital or from elective terminations (Zuckerberg San Francisco General Hospital, San Francisco, CA, USA) and Advanced Bioscience Resources (Alameda, CA, USA). For infection, primary CTBs were isolated from placentas from 3 donors at 16.5-, 20.1-, and 20.2-week gestation as reported [15,18,21,22]. The purity of cells was confirmed by immunostaining with rat monoclonal antibody 7D3, which detects human cytokeratin-7 (cytotrophoblasts) [51] and antibodies to von Willebrand factor (endothelial cells) and vimentin (fibroblasts). Cells were cultured on fibronectin-coated plates in a serum-free medium: DMEM/F12 (1:1) with 2% Nutridoma (Roche Diagnostics, Indianapolis, IN, USA), 1% sodium pyruvate, 1% HEPES, 1% penicillin/streptomycin, and 0.1% gentamycin (UCSF Cell Culture Facility). After 16 h, neutralization assays were performed. Amniotic epithelial cells (AmEpCs) were isolated from amniotic membranes dissected from 21- and 38.6-week placentas [15]. AmEpCs were cultured on fibronectin-coated plates in DMEM/F12 supplemented with 20 ng/mL epidermal growth factor (R&D Systems, Minneapolis, MN, USA), 10% fetal bovine serum (FBS), 1% non-essential amino acids, 55 µM 2-mercaptoethanol (Gibco), and antibiotics and antimycotics (UCSF Cell Culture Facility) as reported [15]. First-passage AmEpCs that were 100% positive by immunostaining with an anti-cytokeratin 19 (CK19) antibody (ProteinTech, Rosemont, IL, USA) were used for experiments. Trophoblast progenitor cells (TBPCs) isolated from the human chorionic membrane (15.6 weeks gestation), provided by O. Genbacev and S. Fisher [52], were cultured on gelatin-coated plates in DMEM/F12 (1:1) supplemented with 10 ng/mL basic fibroblast growth factor (bFGF) (R&D Systems, Minneapolis, MN, USA), 10 µM SB431542 (Tocris Biosciences, Minneapolis, MN, USA), and 10% FBS. TBPCs 100% positive by immunostaining with mAb 7D3 and an antibody against GATA4 (R&D Systems, Minneapolis, MN, USA) were used up to passage 15 for experiments. Human placental fibroblasts (HPFs; 8 weeks gestation), a gift from D. Ilic [53], were grown in DMEM/M199 (4:1) with 1% amino acids and 10% FBS. MRC-5 cells (American Type Culture Collection, Manassas, VA, USA) were cultured in DMEM with 10% FBS. Human adult retinal pigment epithelial (ARPE-19) cells [54,55] were grown in DMEM or DMEM/F12 (1:1) with 10% FBS.

### 2.4. Virus Stocks and Microneutralization Assays in MRC-5 and ARPE-19 Cells

HCMV neutralizing assays were performed in MRC-5 and ARPE-19 cells using strains AD169 (American Type Culture Collection, Manassas, VA, USA), UxCA (kindly provided by Dr. Stuart Adler, Virginia Commonwealth University), and VR1814. Virus stocks were prepared in MRC-5 as follows: Cells were infected with VR1814, harvested along with the supernatant, and flash-frozen when infection reached ~90%. After thawing, cell debris was removed by centrifugation, and the supernatant was aliquoted and stored at −80 °C. Virus titers were measured in a microtitration assay by infecting MRC-5 cells and counting HCMV immediate-early (IE1)-positive cells using a CTL-ImmunoSpot^®^ S6 Analyzer (Cellular Technology, Cleveland, OH, USA). A high-throughput quantitative micro-neutralization assay was performed on both MRC-5 and ARPE-19 cells in 96-well plates. The input virus (approximately 500 PFU/well) was mixed with media containing heat-inactivated serum or serial 3-fold dilutions of antisera and incubated for 1 h at 37 °C. The mixtures were then added to cells for 24 h to allow virus adsorption. HCMV-infected cells were quantified by counting immunostained cells with an anti-HCMV IE1 antibody (MilliporeSigma, Burlington, MA, USA), and an Alexa Fluor 488-conjugated goat anti-mouse Ig (H+L) secondary antibody (Thermal Fisher Scientific, Waltham, MA, USA). Counting was performed using a CTL-ImmunoSpot S6 Analyzer. The serum dilution that produced 50% inhibition for a given test sample was calculated by interpolation using Microsoft Excel.

### 2.5. Virus Neutralization Assays in Primary Cells and Anchoring Villus Explants

For assays using rabbit sera, the following cell types were seeded in 96-well plates and infected at the MOIs indicated: CTBs (MOI 0.08–0.1), AmEpCs (MOI 3.8), TBPCs (MOI 0.1), and HPFs (MOI 0.6). The stock virus was preincubated with media alone (no serum control), heat-inactivated preimmune serum (negative serum), or Cytogam, an HCMV hyperimmune globulin preparation (CSL Behring LLC, King of Prussia, PA, USA), at the indicated final dilutions for 1 h at 37 °C with moderate agitation. Virus–antibody mixtures were added to cells for 2 h in duplicate wells, and cells were washed and further incubated in the medium. At 2 dpi, cells were fixed, permeabilized, and immunostained with a mAb against HCMV IE1 (MilliporeSigma, Burlington, MA, USA). Infected cells were counted, and the percentage of cells infected at each dilution was determined relative to the average number of infected cells in the no-antibody control wells (*n* = 6). Fifty percent neutralizing titers were calculated using Microsoft Excel by the method of Reed and Muench. For assays using rhesus sera, ARPE-19 cells and AmEpCs were seeded on coverslips in 24-well plates. Virus MOI was adjusted to give a total of 600 to 800 infected cells/well by an untreated virus. Percentages of infected cells and 50% neutralization titers were determined as above.

For assays in anchoring villus explants, chorionic villi were dissected from human placentas (8-, 12-, and 14-week gestation), and 20 to 30 villus explants from each placenta were plated on Millicell-CM inserts (0.4 µm pore size, MilliporeSigma, Rocklin, CA, USA) coated with Matrigel in DMEM/F12 (1:1) with 10% FBS, penicillin/streptomycin, and amino acids, conditions that enable the differentiation of villus CTBs into invasive cells [18]. At 20 h after attachment to Matrigel, villus explants were infected overnight with VR1814 (3–4 × 10^6^ PFU), preincubated with control and test sera as described above, washed, and cultured for two more days before fixation and frozen embedding. For quantification of infection in developing cell column CTBs, 1–3 sections of each explant were immunostained with mAb 7D3 and a mAb against HCMV IE1 (MilliporeSigma, Burlington, MA, USA), and all cell columns were imaged on a Leica DMi8 microscope with a Leica DFC9000GT camera controlled by Leica Application Suite X software. Images were imported into Adobe Photoshop and cell columns were identified by cytokeratin staining and cell morphology. Cell column nuclei [DAPI (4′,6-diamidino-2-phenylindole) channel] were quantified in ImageJ using the particle analysis function, and HCMV IE1-positive nuclei were counted manually. The aggregate percentage of cell column CTBs infected in each condition from a placenta (1–2 explants per condition) was determined by combining numbers from all cell columns analyzed.

### 2.6. Antibodies and Reagents

The following antibodies were purchased for immunostaining: Mouse mAbs to HCMV IE1 (MilliporeSigma, Burlington, MA, USA), rabbit anti-vimentin mAb (Abcam, Cambridge, UK), and rabbit polyclonal antibodies to human cytokeratin (CK) 19 (ProteinTech, Rosemount, IL, USA), and von Willebrand factor (ThermoFisher Scientific, Waltham, MA, USA). Rat anti-human cytokeratin mAb (clone 7D3) was a gift from S. Fisher [51].

### 2.7. Immunofluorescence and Imaging

Cells grown on coverslips were fixed with 4% paraformaldehyde and permeabilized with 0.1% Triton X-100. Frozen tissues were cut into 5 µm sections. For double and triple immunostaining, after blocking with 3–5% normal serum matching the secondary antibody source for cells and BSA plus 3–5% normal serum for explants, cells or tissue sections were simultaneously incubated with primary antibodies from different species followed by incubation with secondary antibodies labeled with fluorescein isothiocyanate (FITC) or rhodamine red-X (RRX) (Jackson ImmunoResearch, West Grove, PA, USA). Nuclei were stained with DAPI (Vector Laboratories, Burlingame, CA, USA). Alternatively, cells and tissues were incubated with primary antibodies against cellular proteins overnight, followed by incubation with secondary antibodies, then stained with antibodies to HCMV proteins. Images were obtained using a Leica DMi8 microscope as above. Images of whole explant sections were taken on a Leica M125 stereomicroscope equipped with a Leica MC170HD camera.

## 3. Results

### 3.1. Anti-Pentamer Antibodies from Immunized Rabbits Potently Block Virus Entry into Fibroblasts and Epithelial Cells

**Figure 1:**
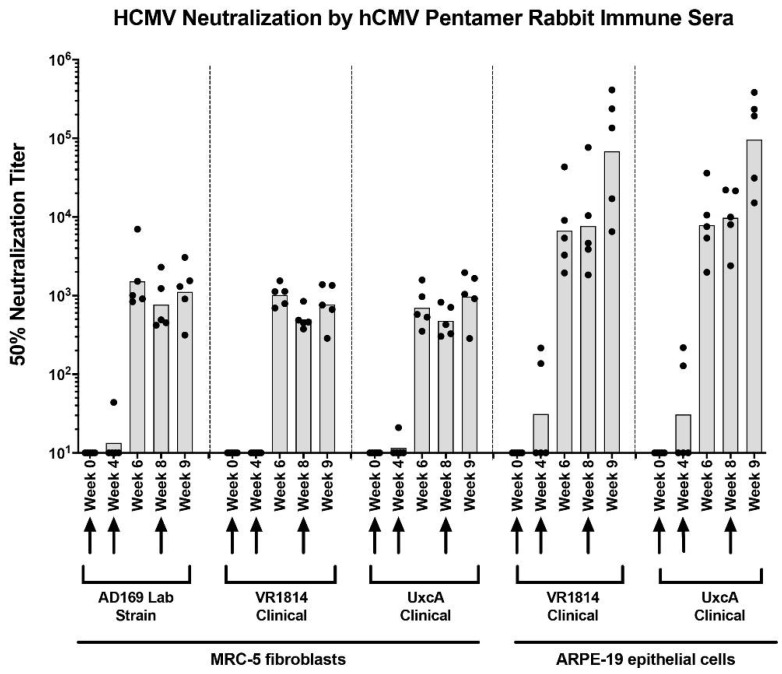
Anti-pentamer rabbit immune sera neutralize HCMV infection of MRC-5 and ARPE-19 cells. Sera were collected at multiple time points from rabbits immunized with RBT pentamer, serially diluted, and tested for their ability to neutralize infection by the laboratory HCMV strain AD169 and the clinical strains VR1814 and UxcA on both MRC5 (fibroblast) and ARPE-19 (epithelial) cells. Fifty percent neutralization titers (NT50s) were determined for each serum sample and plotted as shown. Initial immunization (week 0) was followed by booster shots at weeks 4 and 8, as indicated by arrows. Bars = mean NT50 values, dots = values for individual serum samples.

To determine whether sera from rabbits immunized with recombinant HCMV pentamer (RBT pentamer) contain neutralizing antibodies, we measured neutralizing titers of sera against infection by different HCMV strains on ARPE-19 epithelial cells and MRC-5 fibroblasts (Figure 1). Marked differences in serum neutralizing titers were found between these two cell types. Anti-pentamer antisera blocked infection by clinical strains (VR1814 and UxcA) more potently in ARPE-19 cells than in MRC-5 cells. The average 50% neutralization titers (NT50) in epithelial cells were generally 1–2 logs higher than those in fibroblasts. In addition, the kinetics of neutralizing antibody activities were different between the two cell lines. Neutralizing titers on MRC-5 cells were almost undetectable at week four after initial immunization but increased dramatically by week six, two weeks after the first boost, and sustained similar levels thereafter. For ARPE-19 cells, neutralizing titers were several-fold greater at week four than for MRC-5 cells and continued to increase after the first and second boosts, reaching NT50 values ~10^5^ at week nine, one week after the third boost.

### 3.2. Neutralizing Activities of Anti-Pentamer Rabbit Sera on Placental Cells

**Figure 2:**
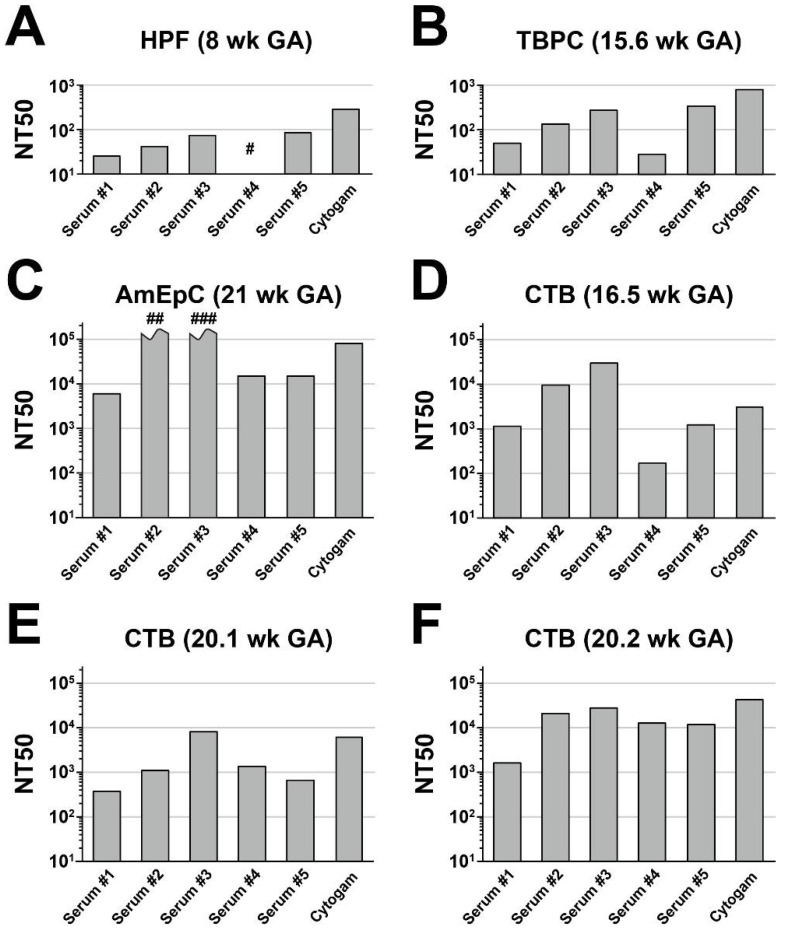
Anti-pentamer rabbit immune sera neutralize HCMV infection of primary placental cell types. Sera from rabbits immunized with RBT pentamer described in Figure 1 were tested for ability to neutralize infection of placental cell types by the clinical HCMV strain VR1814. For comparison, hyperimmune globulin (Cytogam) and non-immune sera were also evaluated. Fifty percent neutralization titers (NT50s) were calculated for each serum sample and plotted for each of the following cell types: (**A**) Human placental fibroblasts (HPFs) from an 8-week-gestation placenta, (**B**) trophoblast progenitor cells (TBPCs) from a 15.6-week-gestation placenta, (**C**) amniotic epithelial cells (AmEpCs) from a 21-week-gestation placenta, (**D**) primary human cytotrophoblasts (CTBs) from a 16-week-gestation placenta, (**E**) CTBs from a 20.1-week-gestation placenta, and (**F**) CTBs from a 20.2-week-gestation placenta. GA, gestational age. #, NT50 < 10. ##, NT50 > 10^5^, NT90 = 4.1 × 10^4^. ###, NT50 > 10^5^, NT90 > 10^5^.

In these experiments, we determined whether RBT pentamer-specific rabbit sera protected against the infection of primary and limited-passage human placental cell types, including HPFs, TBPCs, and AmEpCs. The neutralizing activities of sera from five rabbits at six weeks post-immunization were compared with the neutralizing activities of HIG (Cytogam). All sera exhibited substantially lower neutralizing activities in HPFs (Figure 2A) than in MRC-5 cells (Figure 1), with NT50 values well below 10^2^, more than one log lower than the values measured in MRC5 cells. Serum #4 had very low neutralizing activity, even at the 1:10 dilution (~32% inhibition; NT50 < 10^1^). Cytogam exhibited higher neutralizing activity in these cells, with an NT50 of 3 × 10^2^. Negative control sera showed no appreciable neutralizing activity at the 1:10 dilution (not shown).

Next, we measured neutralizing activities on multipotent TBPCs—precursors of mature syncytiotrophoblasts and CTBs [52]. TBPCs are fully permissive for HCMV infection, but viral entry is independent of the pentameric complex, based on their susceptibility to a UL131A deletion mutant and the finding that anti-gB and anti-gH/gL reduce infection whereas anti-pentamer mAbs targeting pUL130-131 do not [42,43]. All sera had low to moderate neutralizing activities on TBPCs, with NT50 values between 2.9 × 10^2^ and 3.4 × 10^3^, 2–4-fold higher than in HPFs (Figure 2B). Cytogam was more potent, with an NT50 value of 7.9 × 10^3^. Neither of the negative control sera tested exhibited measurable neutralizing activity (not shown).

Next, we carried out neutralizing assays using primary AmEpCs isolated from amniotic membranes. AmEpCs are self-renewing, with stem cell characteristics [56] that support persistent HCMV infection [15]. In cases of congenital infection, HCMV-positive amniotic fluid facilitates diagnosis of transmission [1,9,40,57,58]. Consistent with an earlier study, all sera strongly neutralized infection in AmEpCs in a dose-dependent manner [43]. The two most potent sera (#2 and #3) reduced infection by ~97% and ~85%, respectively, at a 1:100,000 dilution, with NT50 values both exceeding 10^5^ (Figure 2C). NT50 values for the other three sera ranged from 6.4 × 10^3^ to 1.6 × 10^4^. Overall, NT50 values were 2–4 orders of magnitude higher on AmEpCs than on HPFs and TBPCs. By comparison, the NT50 value for Cytogam was somewhat below those of sera #2 and #3. Both negative control sera tested exhibited slight neutralizing activity (~11% inhibition of infection) at the highest concentration (1:10 dilution; not shown). Interestingly, relative activities of individual immune sera were comparable between HPFs and TBPCs, but they did not fully predict their relative activities on AmEpCs.

### 3.3. Anti-Pentamer Rabbit Sera Block Viral Entry into Primary CTBs

We next measured neutralizing activities on primary CTBs isolated from three mid-gestation placentas. After isolation, CTBs initially have an epithelial-like phenotype but subsequently differentiate when cultured on Matrigel, switching to an endothelial-like phenotype [59,60]. All sera neutralized infection dose-dependently, but potencies varied both among sera, as above, but also significantly by cell donor, both in the average magnitude of neutralization and in the relative potencies of individual sera (Figure 2D–F). Average NT50 values by cell donor varied from 2.2 × 10^3^ (20.1-week CTBs; Figure 2E) to 1.5 × 10^4^ (20.2-week CTBs; Figure 2F). Serum #3 exhibited the greatest neutralizing activity on all three donor cells (Figure 2D–F), with NT50 values ranging from 7.5 × 10^3^ to 3.0 × 10^4^. The weakest activity was exhibited by serum #4 on the 16.5-week CTBs (Figure 2D), with an NT50 of 1.7 × 10^2^, two orders of magnitude below that of serum #3 on these cells (NT50 = 3.0 × 10^4^); however, the same serum was among the most potent on the 20.2-week CTBs (Figure 2F) and similar to serum #3 on these cells, with NT50 values of 1.3 × 10^4^ (serum #4) and 2.7 × 10^4^ (serum #3). Cytogam neutralizing activity and activity relative to individual immune sera also varied by the donor, with NT50 values ranging from 3.2 × 10^3^ (Figure 2D) to 4.2 × 10^4^ (Figure 2F).

To assess neutralizing activities in the tissue environment, we performed assays on anchoring villus explants from a placenta at 14 weeks of gestation. In villus explants, CTBs differentiate and invade the Matrigel substrate prior to infection of anchoring villi. We reported that VR1814 replicates in differentiating CTBs in proximal cell columns and reduces outgrowth [61]. mAbs to pUL128-131 of the pentamer potently neutralize infection in cell column CTBs of anchoring villi at ~100-fold lower concentrations than mAbs with targeting specificities for gB and gH [43]. Explants were infected with VR1814-serum mixtures or virus alone and fixed and frozen at 3 dpi, and fixed-frozen sections were immunostained for HCMV IE1 and CK, a CTB marker. Based on the results of primary CTBs from second-trimester placentas, serum dilutions up to 1:1000 were used for explant studies. This dilution achieves 28–63% inhibition of virus infection in primary CTBs (Figure 2D–F). Total and infected CTBs were counted in 371 columns, and the aggregate percentage of infected cells was determined for each condition. In explants infected with VR1814 alone, ~13% of 7D3-positive CTBs were infected, while negative sera exhibited no neutralizing effect (15–30% infected). All three sera tested (#2, #3, and #5) suppressed infection by >99% at the 1:1000 dilution (not shown).

### 3.4. Sera from Rhesus Macaques Immunized with gB and Pentamer Block HCMV Infection of Epithelial Cells and Fibroblasts

**Figure 3:**
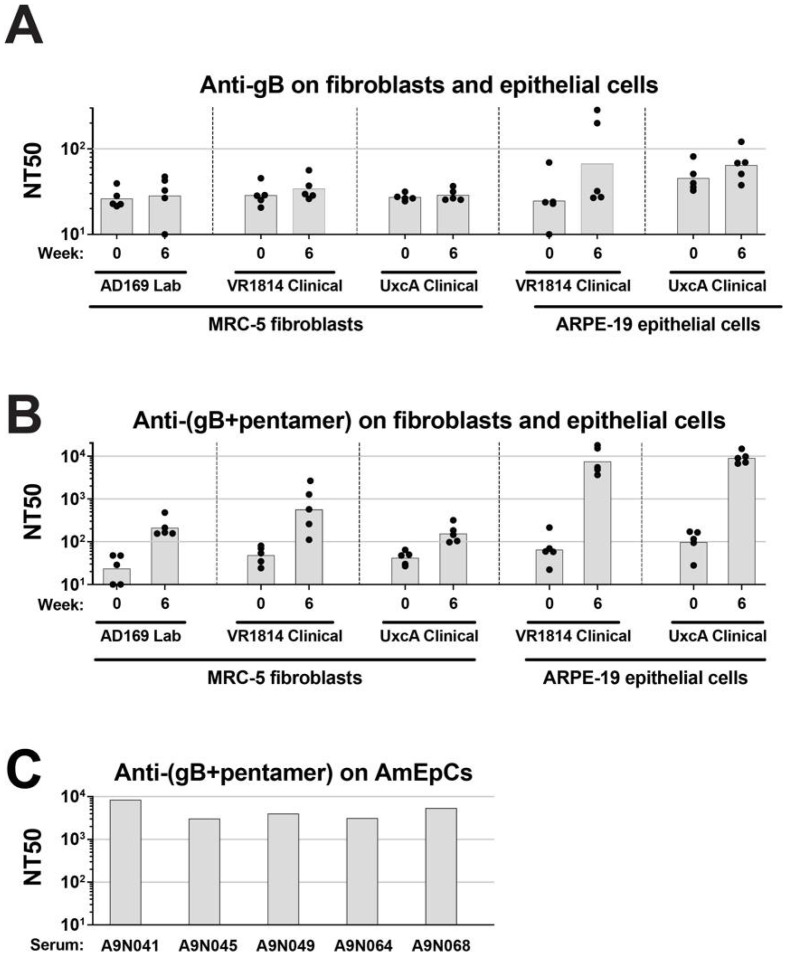
Anti-gB and anti-(gB+pentamer) immune plasma from rhesus macaques neutralize HCMV infection of MRC-5, ARPE-19, and primary amniotic epithelial cells (AmEpCs). (**A**,**B**) Neutralization of infection by VR1814 and UxcA (clinical HCMV strains) and AD169 (laboratory HCMV strain) by sera collected at weeks 0 and 6 from rhesus macaques immunized with either 20 µg of Sino gB (**A**) or 20 µg of RBT gB and 100 µg of RBT pentamer (**B**). Fifty percent neutralization titers (NT50s) are shown for sera from each of five animals in each group (dots), as well as mean NT50 for each condition (bars). (**C**) Neutralization of VR1814 infection of AmEpCs from a 38.6-week-gestation placenta by sera collected at six weeks post-immunization from rhesus macaques immunized with RBT gB + RBT pentamer. NT50 values calculated from the mean neutralization curves (*n* = 2) are shown for each sample.

To evaluate the ability of gB and pentamer to elicit neutralizing antibodies against VR1814 infection in a mammalian model, rhesus macaques were immunized with either recombinant gB produced in mammalian cells (Sino gB) or with recombinant gB and pentamer, both produced in insect cells (RBT gB and RBT pentamer). The gB was not stabilized in the prefusion conformation. Neutralization titers of immunized animals were determined by a microneutralization assay as described (Materials and Methods). All animals in this study were seropositive for rhesus CMV (RhCMV) and had low preexisting titers of cross-neutralizing antibodies against HCMV. As shown in Figure 3A, compared with the sera from week zero (preimmune), sera from week six (after prime and boost) in animals immunized with gB alone showed little increase in neutralization titers in fibroblasts and only a slightly greater increase in neutralization titers in ARPE-19 cells. In contrast, sera from animals immunized with both gB and pentamer achieved ~10-fold higher titers in fibroblasts, and ~100-fold higher titers in epithelial cells (Figure 3B).

Next, we tested the efficacy of the sera (six weeks post-immunization) from rhesus macaques immunized with a combination of gB and the pentamer in neutralizing infection in primary AmEpCs from placentas at delivery (38.6 weeks). All sera at the 1:100 dilution almost completely abolished infection (Figure 3C). NT50 values ranged from 3.1 to 8.6 × 10^3^, with A9N041 exhibiting the greatest activity and A9N045 the lowest.

### 3.5. Antibodies from Rhesus Macaques Immunized with gB and Pentamer Protect against Infection of CTB Cell Columns in Anchoring Villus Explants

**Figure 4:**
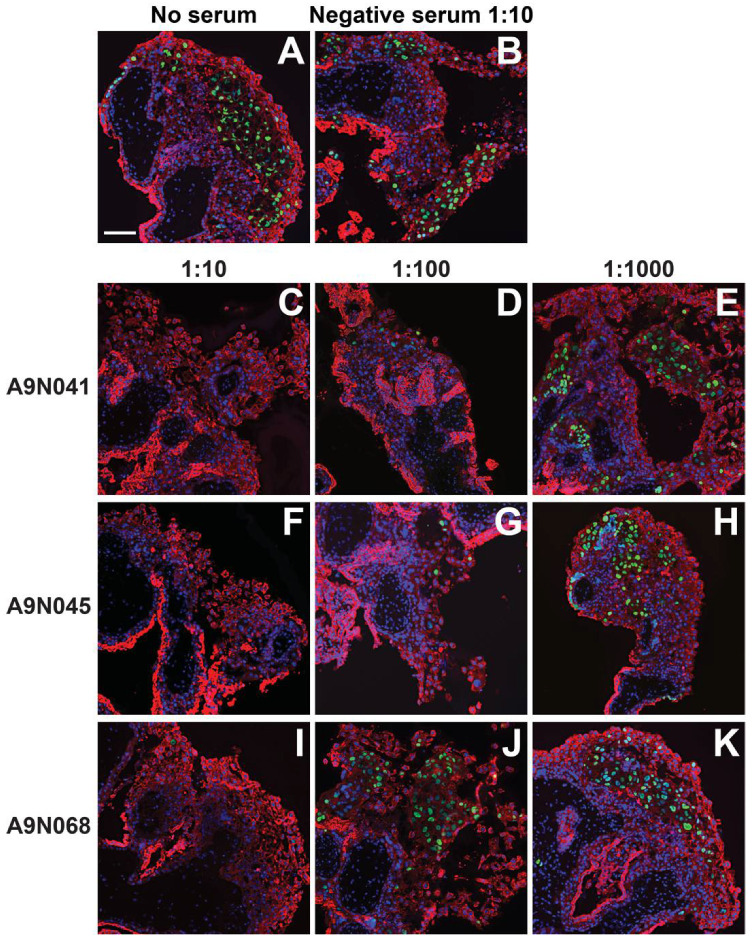
Neutralization of HCMV infection of cell column cytotrophoblasts (CTBs) in anchoring villus explants from an 8-week-gestation placenta by rhesus macaque sera. Neutralization of HCMV infection of cell column CTBs in anchoring villus explants from an 8-week-gestation placenta. Immunofluorescence staining for HCMV IE (green) and cytokeratin (CK) (7D3) (red) in sections of anchoring villus explants from four placentas of different gestational ages infected with VR1814 alone or mixed with selected sera from serial dilution of rhesus macaques immunized with gB and pentamer or not immunized. Quantitative results for experimental conditions are shown in Figure 6. Representative images shown are examples of infection with VR1814 alone (**A**) and infection with VR1814 mixed with negative serum (1:10 dilution; (**B**)) and immune sera A9N041 (1:10; (**C**), 1:100; (**D**) and 1:1000; (**E**)), A9N045 (1:10; (**F**), 1:100; (**G**) and 1:1000; (**H**)) and A9N068 (1:10; (**I**), 1:100; (**J**) and 1:1000; (**K**)). Scale bar = 100 µm. Nuclei were stained with DAPI (blue).

**Figure 5:**
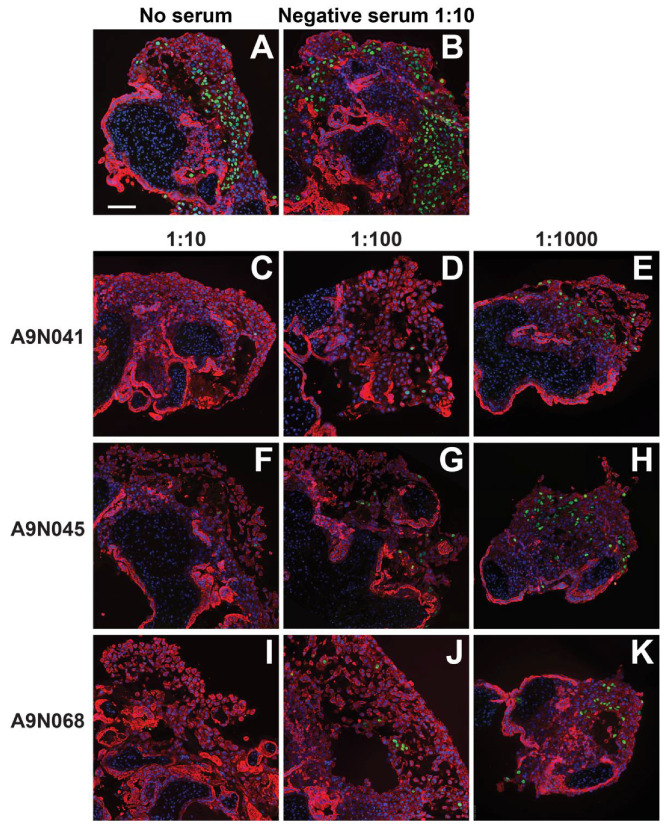
Neutralization of HCMV infection of cell column cytotrophoblasts (CTBs) in anchoring villus explants from a 12-week-gestation placenta by rhesus macaque sera. Neutralization of HCMV infection of cell column CTBs in anchoring villus explants from a 12-week-gestation placenta. Immunofluorescence staining for HCMV IE (green) and cytokeratin (CK) (7D3) (red) in sections of anchoring villus explants from explants infected with VR1814 mixed with selected sera from serial dilutions of sera from rhesus macaques immunized with gB and pentamer or not immunized. Quantitative results for experimental conditions are shown in Figure 6. Representative images shown are examples of infection with VR1814 alone (**A**) and infection with VR1814 mixed with negative serum (1:10 dilution, (**B**)) and immune sera A9N041 (1:10, (**C**); 1:100, (**D**); 1:1000, (**E**)), A9N045 (1:10, (**F**); 1:100, (**G**); and 1:1000, (**H**)) and A9N068 (1:10, (**I**); 1:100, (**J**); and 1:1000, (**K**)). Scale bar = 100 µm. Nuclei were stained with DAPI (blue).

**Figure 6:**
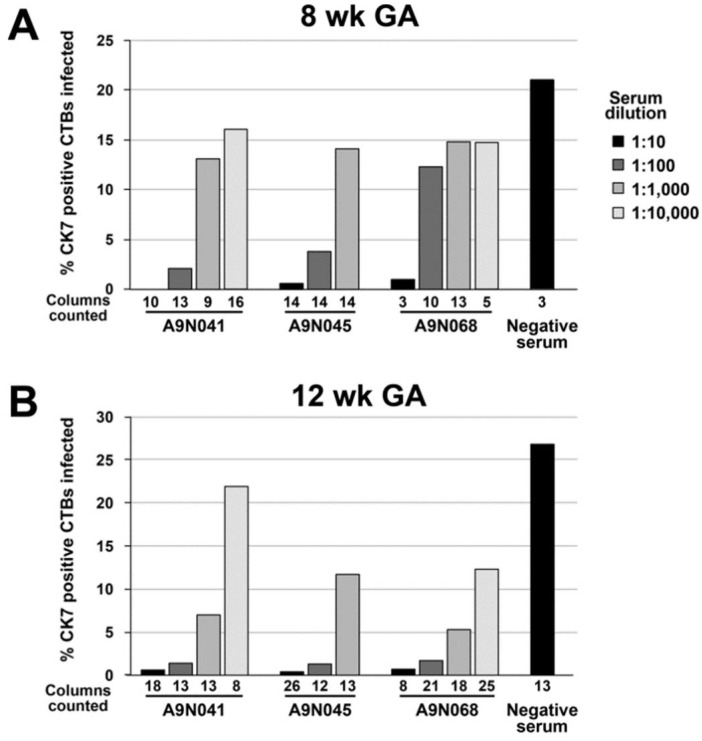
Neutralization of HCMV infection of cell column cytotrophoblasts (CTBs) in anchoring villus explants by rhesus macaque sera. Neutralizing activities on anchoring villus explants from two placentas of 8-week (**A**) and 12-week (**B**) gestational age (GA) corresponding to explants shown in Figure 5 and Figure 6. Explants were infected with VR1814 preincubated with medium alone or with serial dilutions of serum from rhesus macaques immunized with gB and pentamer or not immunized (negative). Both total and infected CTBs were counted in a total of 164 and 223 sections of cell columns from the 8- and 12-week placental explants, respectively. Bars indicate aggregate percentage of cell column CTBs infected (HCMV IE1+) among all cell columns analyzed for each condition. Numbers of cell columns counted are indicated below each bar.

We next assessed the neutralizing activities of selected sera from animals immunized with gB and pentamer on anchoring villus explants from placentas at 8 and 12 weeks of gestation. Up to three independent sections of each of 38 explants were immunostained for CTBs and HCMV IE proteins, and both the number of CTBs and number of infected CTBs were counted in every cell column identified. A total of 376 cell columns from 95 tissue sections were examined, and the aggregate percentages of infected cells were determined for each explant and condition (Figure 4, Figure 5 and Figure 6). In the 8-week-gestation explants infected with VR1814 incubated with preimmune serum (negative serum), 22% of 7D3-positive CTBs were also positive for HCMV IE proteins, whereas preincubation with immune sera from all three animals suppressed infection in a dose-dependent manner (Figure 4A). Relative to VR1814 treated with negative serum, all immune sera suppressed infection of cell column CTBs at dilutions of 1:10 (0–1.2% infected), 1:100 (2.4–12% infected), and 1:1000 (13–15% infected). Similarly, in the 12-week-gestation explants infected with VR1814 treated with negative serum, 27% of CTBs in cell columns were infected (Figure 4B), and all immune sera exhibited high neutralizing activities at dilutions of 1:10 (0.4-0.7% infected), 1:100 (1.3–1.7% infected), and 1:1000 (5.3–11.7% infected). Representative images of tissue sections stained for CTBs and HCMV IE proteins from control and treated explants are shown in Figure 4 (eight-week gestation) and Figure 5 (12-week gestation).

## 4. Discussion

In the present study, we showed that animals immunized with the pentameric complex and gB produce neutralizing antibodies that reduce infection by pathogenic HCMV strains of a broad range of cell types and an ex vivo model of developing early-gestation human placentas. Sera from pentamer-immunized rabbits had neutralizing titers that potently blocked infection by pathogenic viral strains in fibroblast and epithelial cell lines, primary and low-passage placental TBPCs, AmEpCs, CTBs, and differentiating CTBs in anchoring villus explants. Sera from naturally RhCMV infected rhesus macaques subsequently immunized with HCMV gB blocked infection on fibroblast and epithelial cell lines; sera from naturally RhCMV-infected rhesus macaques subsequently immunized with HCMV gB and pentamer had increased neutralizing titers in the cell lines and blocked infection of primary AmEpCs and differentiating CTBs in anchoring villus explants. These results suggest that the combination of the pentameric complex and gB elicits antibodies that provide broad neutralizing activity to the different cell types and thus could reduce viral transmission. These antigens have potential as the basis for a prophylactic vaccine, though the independent role of gB in the neutralization of the virus in placental-derived cells was not demonstrated by these experiments.

Rabbits immunized with a soluble pentamer had higher titers of HCMV neutralizing antibodies on ARPE-19 cells and lower titers on MRC-5 cells and blocked infection of primary placental cell types, most potently AmEpCs. Interestingly, the relative potencies of the sera varied across cell types and among different isolates of CTBs. These results suggest important differences between cells from different tissues and between cells from different individuals. The results underscore the importance of evaluating vaccine-elicited antibodies across a range of primary placental cell types and, when feasible, across multiple donors to accurately model vaccine effectiveness in human populations. Importantly, the rabbit immune sera were able to inhibit infection of both primary CTBs from three different placentas and differentiate CTBs in cell columns of anchoring villus explants. These cells are likely to be the most important conduits of maternal-fetal transmission.

Ongoing efforts to determine naturally protective immune factors have identified maternal immune responses associated with reduced risk of transmission in cases of congenital HCMV infection. These include the early development of neutralizing antibodies to the HCMV glycoprotein pentamer complex [10,62] and increased antibody avidity [8,9,33,36,63,64]. The pentamer has been extensively studied as a vaccine candidate, as the target of the most potent neutralizing antibodies and critical for infection of specialized human cells [28,45,62]. Analysis of sera from HCMV seropositive women showed that neutralizing activities on epithelial cells were considerably higher than on fibroblasts [65]. Antibodies to the pentamer were the majority of the anti-HCMV neutralizing antibody response in HIG [40], and depletion of the pentamer significantly decreased neutralizing activity on epithelial cells [41].

Although the species-specific characteristics of CMV make preclinical testing of HCMV vaccine candidates in animal models a challenge for predicting clinical outcomes, the immunization of rhesus macaques with all five HCMV pentamer subunits resulted in high and sustained neutralizing titers that blocked epithelial and endothelial cell infection and inhibited entry into epithelial cells and fibroblasts [45,46,49]. These results are consistent with the neutralizing antibody responses induced by HCMV during natural infection [27,30,66]. Vaccine-derived HCMV pentamer-specific neutralizing antibodies in animal sera potently prevent virus transfer from endothelial cells to leukocytes and spread in epithelial cells [46], important mechanisms of virus dissemination in vivo, as well as infection of CTBs [43,45]. Inasmuch as CTBs are key targets for infection of the human placenta [18,67], these antibodies could be protective against vertical transmission [19,67,68].

We previously reported that mAbs specific to the pUL128-131 portion of the HCMV pentamer produced by B-cell clones isolated from healthy seropositive donors potently inhibited infection of CTBs and cell-cell spread in anchoring villi [43]. Furthermore, a replication-defective HCMV vaccine elicited neutralizing antibodies as well as CD4^+^ and CD8^+^ T cell responses to multiple viral antigens in rabbits and rhesus macaques [69]. The vaccine also induced long-lived memory B cells at frequencies comparable to those seen in seropositive subjects [70]. Together, our studies provide in vitro and ex vivo evidence for the importance of the pentamer for preventing virus spread in developing human placentas.

Immunization with HCMV gB has been demonstrated to reduce horizontal transmission of HCMV [71]. In phase II trials, a monomeric recombinant gB subunit vaccine candidate with M59 adjuvant demonstrated ~50% efficacy in seronegative women of reproductive age against HCMV primary infection [71,72] and significantly reduced viremia and shortened antiviral treatment in solid organ transplant recipients [73]. Thus, eliciting antibody responses to multiple epitopes of gB and the pentamer may contribute to potential vaccine-mediated protection against congenital transmission. The natural conformation of gB within the viral envelope is a trimer, and some prefusion gB must be present on the virion surface to mediate fusion of the viral envelope and a cellular membrane during entry. In the present study, we immunized rhesus macaques with a form of recombinant gB that was trimeric but not stabilized in the prefusion conformation [74]. Therefore, this gB immunogen is not likely to have the conformation of gB on virions or the surfaces of HCMV-infected cells.

There is evidence that cellular immune responses that include the early development of HCMV-specific CD4^+^ and CD8^+^ T cells can contribute to reducing the transmission of the virus from pregnant women to the fetus after primary infection in the first trimester [11,12]. In human pregnancy, the basal decidua of the uterine wall contains immune cells, including natural killer cells, macrophages, and T cell subsets, which could protect the placenta and fetus [75]. We reported that HCMV replicates in the tissue environment at the uterine–placental interface that serves as a local source of virus spread to the anchoring placenta [19,75]. Specifically, the virus replicates in decidual stromal cells and epithelial cells of endometrial glands. The effector memory CD8^+^ T cells expand and cluster at sites of infection and adjacent to the epithelium of glands and produce IFN-γ, suggesting a central role in controlling early responses to infection in the decidua of seropositive women. Interestingly, long-lived “memory-like” NK cells have been identified in HCMV-infected individuals, but little is known about their generation and density in tissue [76]. These cells exhibit preferential expansion in response to HCMV-infected cells in an antibody-dependent manner, suggesting they could contribute to the control of viral replication in tissue. Intramuscular vaccination followed by electroporation of mice with enhanced DNA plasmid vectors encoding gH/gL or UL128/UL130/UL131A induced robust CD8^+^ T cell responses [77]. Furthermore, mice immunized with lipid nanoparticles encapsulating mRNA encoding the pentameric complex and gB and a second mRNA vaccine expressing the immunodominant CMV T cell antigen pp65 elicited broad and durable neutralizing antibodies as well as strong pentameric complex-specific T cell responses. These results suggest that the addition of pentamer to a T cell antigen generates a more robust combined humoral and cell-mediated immune response that may confer greater protection [78].

In summary, we have shown that the immunization of animals with the HCMV pentamer and combined immunization with pentamer and gB elicit robust neutralizing activity against infection of a broad array of human cell types, including CTBs and other placental cells that may enable vertical transmission. The recent success of RNA vaccines against the severe acute respiratory syndrome coronavirus-2 (SARS-CoV-2) spike glycoprotein to prevent COVID-19 disease [79], which elicit neutralizing titers comparable to natural infection and cellular immune responses with specific CD8^+^ and CD4^+^ T cell expansion [80,81], has raised interest in using this technology in vaccines against additional targets. Thus, HCMV pentamer and gB antigens, whether produced as subunits or encoded by formulated RNA, hold promise for a new generation of HCMV vaccine candidates.

## Data Availability

Not applicable.
